# Sensory Characteristics of Combinations of Phenolic Compounds Potentially Associated with Smoked Aroma in Foods

**DOI:** 10.3390/molecules23081867

**Published:** 2018-07-26

**Authors:** Hongwei Wang, Edgar Chambers, Jianquan Kan

**Affiliations:** 1 College of Food Science, Southwest University, Chongqing 400715, China; wanghw_1978@swu.edu.cn; 2 Center for Sensory Analysis and Consumer Behavior, Kansas State University, Manhattan, KS 66502, USA

**Keywords:** phenolic combinations, odor profile, smoked aroma

## Abstract

The sensory characteristics of phenolic compounds combinations were evaluated. A highly trained descriptive panel evaluated combinations of chemicals (two chemicals at a time) containing either one smoky aroma and one non-smoky aroma chemical compound, two smoky aroma chemicals, or two non-smoky aroma chemicals. The non-smoky compounds had been associated with smoke aroma in other studies, but were not found to be smoky when tested individually. Smoked flavor characteristics and intensities were changed significantly when two phenolic compounds were combined. Non-smoky phenolic compounds often contributed the smoked flavor when combined with one smoky phenolic compound or another non-smoky phenolic compound. It is necessary to understand the sensory characteristics of compound combinations as well as individual compounds.

## 1. Introduction

Since ancient times smoke was used as a food preservation method. Currently, the aim of smoke processing, besides extending shelf-life, is mainly to develop the unique smoked flavor [1,2]. Smoked flavor is not a single smoky characteristic, but rather includes fourteen terms such as smoky, ashy, woody, musty/dusty, musty/earthy, burnt, acrid, pungent, petroleum-like, creosote/tar, cedar, bitter, metallic and sour [3].

Many phenolic compounds have been found as part of smoked aroma characteristics when separated by gas chromatography-olfactometry methods and gas chromatography-mass spectrometry [4,5]. Several phenolic compounds, such as 2,6-dimethoxyphenol, 4-ethyguaiacol, thymol, guaiacol, carvacrol, were described as chemical compounds potentially associated with smoky aroma in foods by highly trained sensory panelists [1]. Various phenolic compounds were used to develop the chemical standard references of smoke flavourings [6]. Those chemicals included pyrocatechol, 3-methoxypyrocatechol, 1,2-dimethoxybenzene, *p*-cresol, thymol, guaiacol, eugenol, *m*-cresol, *o*-cresol and isoeugenol. Smoke taint in wine was associated with volatile phenols and glycoconjugated phenols [7,8,9,10,11,12,13,14] and the removal of smoke-derived volatile phenols were used to ameliorate smoke taint in wine [15]. Of course, the odor character of compounds can change at different concentrations [16,17]. Sensory characteristics of a set of individual phenolic compounds were evaluated using a professionally developed set of sensory attributes [18]. Among the nineteen phenolic compounds, only 2,6-dimethylphenol was not present in smoky characteristics in that study. However, these chemical compounds do not occur individually in volatile fractions of smoked products [19,20,21,22].

Combinations of volatiles may yield different flavors than those expected from individual compounds [23]. Flavour perception is characterised by complex interactions between physicochemical processes and (bio) chemical, physiological and behavioural phenomena [24]. Zou and Buck [25] proposed that cortical neurons require combinations of receptor inputs for activation and that merging the receptor codes of two odorants provides novel combinations of receptor inputs that stimulate neurons beyond those activated by the single odorants. In that study, the mixture of combined major odorants was perceived to possess more resemblance to the characteristic odor of aqueous soy protein isolates than any individual odorant [26]. Bott and Chambers [27] found overall beany character in various combinations from each grouping, including combinations of chemicals that alone were not considered beany. Researchers of smoky chemical compounds [18] have suggested that combinations of compounds need to be assessed in order to better understand the potential of those compounds to impact smokiness.

In this research, combinations of two phenolic compounds were studied in order to examine the effect of combining phenolic chemicals associated with smoked aroma in foods. The objectives of this study were to: (1) determine how combining two smoky chemicals affects smoked characteristics and intensity; (2) determine how combining one non-smoky individual compound with one smoky compound affects intensity or perception of smoked character; and (3) determine if combining two non-smoky phenolic chemicals produces smoky odor.

## 2. Results and Discussion

The smoked aroma characteristics were defined and referenced by Jaffe et al. [3]. The list of sensory attributes (Table 1) used in this research, included smoky, ashy, woody, musty/dusty, musty/earthy, burnt, acrid, pungent, petroleum-like, creosote/tar or cedar. A chemical attribute was added to the lexicon during this testing because panelists noted there was a chemical-like note closely associated with smoke that was part of the overall smokiness in the combinations. Other researchers have noted that lexicons for evaluation are not static and can be adjusted depending on the context of the research [28].

### 2.1. Combinations of Two Smoky Phenolic Compounds

Two compounds, 100 ppm 4-ethylguaiacol and 100 ppm 2,6-dimethoxyphenol were used to combine with other smoky chemicals. Those compounds were chosen because the ethylguiacol compound had a unidimensional odor that was only smoky with no other odor character at 100 ppm and the dimethoxyphenol compound showed a multidimensional odor with smoky and other odors, such as woody, at 100 ppm [18]. A total 16 combinations of two smoky compounds were evaluated (Table 2 and Table 3).

The smoky character remained in all combinations of the two smoky compounds. That is not the case for all types of odors, however. In a study of beany odor, some combinations of two beany compounds did not retain beany odor characteristics [27]. Most combinations resulted in smoky aroma intensity that was lower than the intensity of the smoky phenolic compounds alone, suggesting some suppression of smoky odor by other competing aroma characteristics. Four combinations, 100 ppm 4-ethylguaiacol and 100 ppm *m*-cresol; 100 ppm 4-ethylguaiacol and 100 ppm guaiacol; 100 ppm 2,6-dimethoxyphenol and 10 ppm *m*-cresol; 100 ppm 2,6-dimethoxyphenol and 100 ppm *m*-cresol, resulted in smoky aroma intensity that was higher than individual chemicals. The increase in scores for smoky intensity were small (0.5 or 1.0) compared with the higher smoky intensity of individual chemicals. Three combinations had equal smoky aroma intensity with the higher of the individual chemicals.

When different concentrations of the same chemical were combined with 100 ppm 4-ethylguaiacol or 100 ppm 2,6-dimethoxyphenol, the higher concentration mostly presented higher smoky intensity. This shows the expected psychophysical relationship of perceived intensity to actual intensity. However, there were two combinations, 100 ppm 2,6-dimethoxyphenol combined with different concentrations of 4-ethylguaiacol and 100 ppm 4-ethylguaiacol combined with different concentrations of *p*-cresol, in which the higher concentration present lower smoky intensity. This suggests suppression either by chemical interaction or through higher levels of non-smoky intensities to the perception of smoky. Other authors also point to the complexity of odor impacts when guaiacol is present in complex mixtures [28].

Three combinations maintained their woody characteristic; all eight combinations included 100 ppm 4-ethylguaiacol. Four other phenolic individual chemical compounds presented woody characteristics, but when combined with 100 ppm 4-ethylguaiacol, only two combinations maintained the woody characteristic.

Many additional smoked characteristics, such as ashy, woody, burnt, acrid, pungent, petroleum-like, were presented in the combinations. For example, both 100 ppm 2,6-dimethoxyphenol and 1 ppm 4-ethylguaiacol were only described as smoky when presented individually, but, woody was added to the description when two chemicals were combined. Kelly and Zerihun [7] showed that guaiacol and syringol (2,6-dimethoxyphenol) often appeared together in smoky odors, and both increased smokiness. Hwoever, those compounds were found differentially in various types of wood and, thus, perhaps contributed to smokiness in different ways.

Some individual odor attributes were not present in the combinations. For instances, 100 ppm *p*-cresol presented smoky, musty/dusty, petroleum-like attributes when tested individually, but the combination of 100 ppm *p*-cresol and 100 ppm 2,6-dimethoxyphenol only showed the overall smoky attribute.

### 2.2. Combinations of One Smoky and One Non-Smoky Phenolic Compound

100 ppm 4-ethylguaiacol and 100 ppm 2,6-dimethoxyphenol were combined with non-smoky chemicals. A total 11 combinations of one smoky and one non-smoky chemical were evaluated. The combinations of smoky with non-smoky phenolic compounds produced more smoked aroma characteristics than the individual chemicals (Table 4), indicating the potential of a non-smoky chemical to enhance the character and intensity of smoky ones. This phenomenon has been discussed before related to other chemical compounds (**23**,**27**). For smoky aroma, phenols such as guaiacol compounds and syringol often were found with other non-phenolic compounds in smoky odors from fires and those combinations were able to differentiate among various sources of smoke [7,13].

The smoky character of 100 ppm 2,6-dimethoxyphenol and 100 ppm 4-ethylguaiacol remained in all combinations. Although 2,6-dimethylphenol was not associated with smoked characteristics, different concentrations (10 ppm and 100 ppm) of 2,6-dimethylphenol raised the smoky aroma intensity of the combinations with 100 ppm 2,6-dimethoxyphenol or 100 ppm 4-ethylguaiacol. 10 ppm 2,6-dimethylphenol raised smoky aroma intensity of 100 ppm 4-ethylguaiacol higher than 100 ppm 2,6-dimethylphenol. However, 100 ppm 2,6-dimethylphenol raised higher smoky aroma intensity of 2,6-dimethoxyphenol more than 10 ppm 2,6-dimethylphenol. The combination of 100 ppm 4-ethylguaiacol and 10 ppm 2,6-dimethylphenol presented the highest smoky intensity in the study, which was 4.0 on a 15-point scale. 10 ppm 4-allyl-2,6-dimethylphenol did not change its smoky intensity when combined with 100 ppm 2,6-dimethoxyphenol and 100 ppm 4-ethylguaiacol. 1 ppm 2,5-dimethylphenol did not change the smoky intensity when combined with 100 ppm 2,6-dimethoxyphenol, and showed decreased smoky intensity when combined with 100 ppm 4-ethylguaiacol. One ppm eugenol decreased its smoky intensity when combined with 100 ppm 2,6-dimethoxyphenol and 100 ppm 4-ethylguaiacol. One ppm 2,6-dimethoxyphenol also decreased the smoky intensity of 100 ppm 4-ethylguaiacol from 3 to 1.5 showing that sensory suppression as well as enhancement can occur when chemicals are combined.

The woody character of 100 ppm 4-ethylguaiacol remained in most combinations, except the combination with 100 ppm 2,6-dimethylphenol. The woody intensities were described as 1.5 or 2 on a 15-point scale. Although 100 ppm 2,6-dimethoxyphenol did not present woody character, most combinations with non-smoky chemicals had a woody term. The woody intensities were described as 1.5 on a 15-point scale.

Many additional smoked characteristics, such as ashy, woody, burnt, acrid, pungent, musty/dusty, were presented in the combinations.

### 2.3. Combinations of Two Non-Smoky Phenolic Compounds

100 ppm 2,6-dimethylphenol and 1 ppm 2,6-dimethoxyphenol were used to combine with other non-smoky compounds. Neither 2,6-dimethylphenol nor the other compounds were associated with smoked characteristics during screening at the levels used in this study. However, the compounds have been found in smoky odors. A total of seven combinations of two non-smoky phenolic compounds were evaluated. Five of the combinations were identified as now having smoked aroma characteristics (Table 5). These combinations had a smoky odor ranging from 2.5 to 3.0 on a 15-point scale. The combination of non-smoky compounds to give smoky compounds can result from the two compounds forming other compounds, the ability of the two compounds to activate neurons producing novel odors [25], or causing the suppression of other odors so that the smokiness of the individual compounds is highlighted. The similar results were found in some combinations made of two non-beany compounds [27]. Two combinations, 1 ppm 2,6-dimethoxyphenol combined with 1 ppm eugenol and 10 ppm 4-allyl-2,6-dimethoylphenol, did not show any odor suggesting that the two individual compounds suppressed each other. Another two combinations containing 1 ppm 2,6-dimethoxyphenol presented many odor characteristics related to smoked flavor, such as smoky, woody, ashy, musty/dusty, burnt, acrid and pungent. All combinations containing 100 ppm 2,6-dimethylphenol had a smoky odor and other characteristics associated with smoked flavor, such as woody, ashy, musty/dusty, musty/earthy, burnt, acrid, pungent and petroleum-like. Although phenols were higher in smoked than non-smoked dry-cured hams [29], aromatic hydrocarbons and acids also were higher in the smoked hams. That suggests that those two types of compounds, which are not necessarily smoky individually, play a role in smokiness and could combine to provide additional smoky flavor to foods.

## 3. Materials and Methods

### 3.1. Panelists

Highly trained panelists from the Sensory Analysis Center at Kansas State University (Manhattan, KS, USA) took part in this research. All the panelists had completed 120 h of sensory descriptive training and each had more than 1000 h of testing experience, including smoked products [3] and various concentrations of phenol compounds [18]. Each panelist had a broad background of experience in odor description and evaluation. Profiles from this and similar panels have been used extensively in sensory testing of chemicals and food and other products (e.g., [16,17,18,27,30,31,32,33,34,35,36]).

### 3.2. Chemicals

All chemicals used had been evaluated previously by a descriptive panel and classified as having smoky or non-smoky characteristics [18] as individual chemicals at various concentrations. Smoky chemicals were those that possessed a smoky attribute. Some smoky chemicals also possessed other sensory attributes that are associated with smoked odors such as woody, ashy, musty/dusty, acrid, pungent, and petroleum-like. Compounds chosen for this study were selected on the basis of the previous study [18] and either one or two levels were selected depending on the findings in that study.

Individual phenolic compounds with smoky characteristics used in this research were: 100 ppm 2,6-dimethoxyphenol, 1 ppm 4-ethylguaiacol, 100 ppm 4-ethylguaiacol, 10 ppm *m*-cresol, 100 ppm *m*-cresol, 10 ppm *p*-cresol, 100 ppm *p*-cresol, 1 ppm guaiacol, 100 ppm guaiacol, 10 ppm carvacrol. Only 2,6-dimethylphenol was not associated with smoked characteristics by screening. Some phenol compounds at low concentrations were used as non-smoky chemicals. Individual non-smoky phenolic compounds included in this research were 100 ppm 2,6-dimethylphenol, 1 ppm 2,6-dimethoxyphenol, 1 ppm eugenol, 10 ppm 4-allyl-2,6-dimethoylphenol, 1 ppm 2,5-dimethylphenol. All chemicals were purchased from Fisher Scientific Co. (Fair Lawn, NJ, USA).

### 3.3. Sample Preparation

To prepare stock solutions for the study, all chemicals were diluted in propylene glycol (Sigma-Aldrich Co., St. Louis, MO, USA) [16,17,18,27]. To prepare combinations, double the concentration of each individual chemical final dilution was prepared. To prepare the samples for use, equal milliliters of each stock chemical dilution were combined to give the final dilution. Because two chemical dilutions were combined to produce the final chemical concentration, the concentrations were half of each of the original chemical dilution used [27]. For example, combining 1 mL of 200 ppm 4-ethylguaiacol and 1 mL of 20 ppm m-cresol produced a final chemical dilution of 100 ppm 4-ethylguaiacol and 10 ppm *m*-cresol. To deliver the chemicals, a fragrance strip was dipped to a 1.25 cm depth of a specified chemical solution and placed into a 20-mL capped, coded glass tube. Chemical solutions and the fragrance strip preparation were completed approximately 24 h prior to testing.

### 3.4. Evaluation Procedure

The odor profiles of combinations of two phenolic compounds were evaluated during five 1.5 h sessions. Six or seven combinations and all references were presented in each session. All samples were coded with 3-digit numbers and the order in which chemicals were evaluated was randomized. Testing was conducted at the Center for Sensory Analysis and Consumer Behavior at Kansas State University in a room specially designed for sensory testing. The room is temperature, humidity and lighting controlled and the air turnover in the room is double that used for standard office spaces in order to reduce odor buildup.

A modified sensory profile method as described previously [16,18,27] was used to generate an odor profile of the combinations of phenolic compounds. Panelists evaluated each phenol combination by taking quick sniffs from the fragrance testing strips. Each panelist examined samples individually, and then as a group, determining the odor profile of the combination of two phenol compounds. For profiling, a 15-point intensity scale was used, where 1 represents “just recognizable” and 15 represents “extremely intense”.

To reduce carryover 5 to 10 min elapsed between sniffing different combinations. Panelists stopped testing anytime carryover from one sample to another could not be avoided. Fresh air and sniffs of a warm, wet towel were used to cleanse the nasal passages between samples.

## 4. Conclusions

Sensory characters of most combinations with two phenolic compounds were changed from those smelled individually. Combinations did not show particular consistency in their effects. Thus, additional research will be needed to understand the chemical structure and neuronal interface of the combinations.

When two smoky phenolic compounds were combined, the smoky character remained in all combinations, but the smoky intensity changed irregularly. Some additional smoked characteristics, such as ashy, woody, burnt, acrid, pungent, petroleum-like became apparent in the combinations while some individual odor attributes did not remain in the combinations.

When 100 ppm 4-ethylguaiacol or 100 ppm 2,6-dimethoxyphenol combined with non-smoky phenolic compounds, smoky character was maintained in all samples, but the intensity changed irregularly. More smoked aroma characteristics of the combinations were determined than when 100 ppm 4-ethylguaiacol or 100 ppm 2,6-dimethoxyphenol was tested individually.

2,6-Dimethylphenol did not show smoked odor individually, but contributes to smoked odor characteristics when combined with another non-smoky phenolic compounds at low concentrations. About half of the combinations containing 1 ppm 2,6-dimethoxyphenol were not determined to have any odor character and the other half presented many odor characteristics related to smoked flavor, such as smoky, woody, ashy, musty/dusty, burnt, acrid and pungent.

The contribution of non-smoky compounds indicates the complexity of the smoked characters and indicates the need for understanding combinations of odors as well as individual compounds. In actual use, multiple compounds are present and may enhance, suppress, or have no effect on the aroma and, thus, flavor characteristics. This points to the need for large studies to determine the principles on which odors change perception when they are combined. It is clear that simple categorization of compounds based solely on type (i.e., phenolic compounds) is insufficient to provide a “rule” for understanding the sensory perception of combinations of chemical compounds.

## Figures and Tables

**Table 1:** List of sensory attributes associated with smoked aroma characteristics *.

Attribute	Attribute	Attribute
Smoky	Ashy	Woody
Musty-Dusty	Musty/Earthy	Burnt
Acrid	Pungent	Petroleum-like
Creosote/Tar	Cedar	

* 11 attributes from Jaffe et al. [3] that are smoky aroma active.

**Table 2:** Odor profiles * of combinations of 100 ppm 4-ethylguaiacol and another smoky phenolic compound.

Smoky Phenolic Compound 1		Smoky Phenolic Compound 2		Combinations of Two Smoky Phenolic Compounds	
4-ethylguaiacol at 100 ppm		*m*-cresol at 10 ppm			
Smoky	3	Smoky	2	Smoky	1.5
Woody	2	Woody	1.5	Chemical	3
		Musty/Dusty	1.5		
		Petroleum-like	2		
		*m*-cresol at 100 ppm			
		Smoky	2.5	**Smoky**	**3.5**
		musty/Dusty	2.0	Ashy	1.5
		Petroleum-like	2.0	Woody	2
		Chemical	2.5	Chemical	3.5
		*p*-cresol at 10 ppm			
		Smoky	1.5	Smoky	3
		Musty/Dusty	1.5	Ashy	2
				Burnt	1.5
				Acrid	1.5
				Pungent	1.5
				Petroleum-like	2
				Chemical	4
		*p*-cresol at 100 ppm			
		Smoky	1.5	Smoky	1.5
		Musty/Dusty	1.5	Petroleum-like	1.5
		Petroleum-like	1.5	Chemical	2
		guaiacol at 1 ppm			
		Smoky	2.5	Smoky	2.5
		Woody	2	Woody	2
				Musty/dusty	1.5
				Chemical	3
		guaiacol at 100 ppm			
		Smoky	3.5	**Smoky**	**4**
		Woody	3.0	Ashy	2
		Musty/Dusty	2.0	Woody	1.5
		Petroleum-like	1.5	Musty/dusty	1.5
				Pungent	1.5
				Chemical	2
		carvacrol at 10 ppm			
		Smoky	2.5	Smoky	2
		Woody	2.5	Musty/earthy	2
		Musty/Dusty	2.0	Petroleum-like	2
		Pungent	2.0		
		Cedar	2.5		
		2,6-dimethoxyphenol at 100 ppm			
		Smoky	2.5	Smoky	2
				Woody	1.5
				Musty/dusty	1.5

* Intensity is based on a 15-point scale where 1 is “just recognizable” and 15 is “extremely intense”; The smoky intensity of combonation which was higher than individual chemicals is in bold.

**Table 3:** Odor profiles * of combinations of 100 ppm 2,6-dimethoxyphenol and another smoky phenolic compound.

Smoky Phenolic Compound 1		Smoky Phenolic Compound 2		Combinations of Two Smoky Phenolic Compounds	
2,6-dimethoxyphenol at 100 ppm		*m*-cresol at 10 ppm			
Smoky	2.5	Smoky	2	**Smoky**	**3**
		Woody	1.5	Ashy	2
		Musty/Dusty	1.5	Burnt	2
		Petroleum-like	2	Acrid	2.5
				Petroleum-like	2
		*m*-cresol at 100 ppm			
		Smoky	2.5	**Smoky**	**3.5**
		musty/Dusty	2.0	Ashy	1.5
		Petroleum-like	2.0	Acrid	2
		Chemical	2.5		
		*p*-cresol at 10 ppm			
		Smoky	1.5	Smoky	1.5
		Musty/Dusty	1.5	Acrid	2
				Pungent	1.5
		*p*-cresol at 100 ppm			
		Smoky	1.5	Smoky	2
		Musty/Dusty	1.5		
		Petroleum-like	1.5		
		guaiacol at 1 ppm			
		Smoky	2.5	Smoky	2.5
		Woody	2	Acrid	1.5
		guaiacol at 100 ppm			
		Smoky	3.5	Smoky	3
		Woody	3.0	Woody	2
		Musty/Dusty	2.0	Acrid	1.5
		Petroleum-like	1.5		
		carvacrol at 10 ppm			
		Smoky	2.5	Smoky	2
		Woody	2.5	Ashy	1.5
		Musty/Dusty	2.0		
		Pungent	2.0		
		Cedar	2.5		
		4-ethylguaiacol at 1 ppm			
		Smoky	1.5	Smoky	2.5
				Woody	1.5

* Intensity is based on a 15-point scale where 1 is “just recognizable” and 15 is “extremely intense”; The smoky intensity of combonation which was higher than individual chemicals is in bold.

**Table 4:** Odor profiles * of combinations of one smoky and one non-smoky phenolic compound.

4-Ethylguaiacol at 100 ppm and 2,6-Dimethylphenol at 10 ppm		2,6-Dimethoxyphenol at 100 ppm and 2,6-Dimethylphenol at 10 ppm	
Smoky	4.0	Smoky	3.0
Woody	2.0	Ashy	1.5
Musty/dusty	3.0	Woody	1.5
		Musty/dusty	2.5
		Chemical	2.0
4-Ethylguaiacol at 100 ppm and 2,6-Dimethylphenol at 100 ppm		2,6-Dimethoxyphenol at 100 ppm and 2,6-dimethylphenol at 100 ppm	
Smoky	3.5	Smoky	3.5
Musty/earthy	2.5	Ashy	1.5
Burnt	2.5	Musty/dusty	2.0
Acrid	2.0	Burnt	1.5
Pungent	2.0	Acrid	1.5
		Pungent	1.5
		Chemical	2.0
4-Ethylguaiacol at 100 ppm and eugenol at 1 ppm		2,6-Dimethoxyphenol at 100 ppm and eugenol at 1 ppm	
Smoky	2.5	Smoky	2.0
Ashy	1.5	Woody	1.5
Woody	1.5		
Musty/dusty	2.0		
Chemical	2.0		
4-Ethylguaiacol at 100 ppm and 4-allyl-2,6-dimethoylphenol at 10 ppm		2,6-Dimethoxyphenol at 100 ppm and 4-allyl-2,6-dimethoylphenol at 10 ppm	
Smoky	3.0	Smoky	2.5
Woody	2.0	Ashy	1.5
Musty/earthy	2.0	Woody	1.5
Pungent	2.0		
Chemical	2.0		
4-Ethylguaiacol at 100 ppm and 2,5-dimethylphenol at 1 ppm		2,6-Dimethoxyphenol at 100 ppm and 2,5-dimethylphenol at 1 ppm	
Smoky	2.5	Smoky	2.5
Ashy	2.0	Ashy	1.5
Woody	2.0	Woody	1.5
Musty/dusty	2.0	Musty/dusty	1.5
4-Ethylguaiacol at 100 ppm and 2,6-Dimethoxyphenol at 1 ppm			
Smoky	1.5		
Woody	2.0		
Chemical	2.0		

* Intensity is based on a 15-point scale where 1 is “just recognizable” and 15 is “extremely intense”.

**Table 5:** Odor profiles * of combinations of two non-smoky phenolic compounds.

2,6-Dimethylphenol at 100 ppm and 4-Allyl-2,6-Dimethoylphenol at 10 ppm		2,6-Dimethylphenol at 100 ppm and 2,6-Dimethoxyphenol at 1 ppm	
Smoky	2.5	Smoky	2.5
Ashy	1.5	Woody	1.5
Musty/Dusty	1.5	Musty/Earthy	3
Petroleum-like	1.5	Pungent	1.5
Chemical	1.5	Chemical	2
2,6-Dimethylphenol at 100 ppm and 2,5-dimethylphenol at 1 ppm		2,6-Dimethoxyphenol at 1 ppm and 2,5-dimethylphenol at 1 ppm	
Smoky	3	Smoky	3
Ashy	1.5	Ashy	1.5
Musty/Dusty	2.5	Musty/Dusty	1.5
Burnt	1.5	Burnt	1.5
Acrid	1.5	Acrid	2
Pungent	1.5	Pungent	1.5
2,6-Dimethylphenol at 100 ppm and eugenol at 1 ppm			
Smoky	3		
Woody	2		
Musty/Dusty	2		

* Intensity is based on a 15-point scale where 1 is “just recognizable” and 15 is “extremely intense”.

## References

[1] Chambers D.H., Chambers E., Seitz L.M., Sauer D.B., Robinson K., Allison A.A. (1998). Sensory characteristics of chemical compounds potentially associated with smoky aroma in foods. Dev. Food Sci..

[2] Vidal N.P., Goicoechea E., Manzanos M.J., Guillén M.D. (2017). Effect of smoking using smoke flavorings on several characteristics of farmed sea bass (*Dicentrarchus labrax*) fillets and on their evolution during Vacuum-Packed storage at refrigeration temperature. J. Food Process. Preserv..

[3] Jaffe T.R., Wang H., Chambers E. (2017). Determination of a lexicon for the sensory flavor attributes of smoked food products. J. Sens. Stud..

[4] Varlet V., Knockaert C., Prost C., Serot T. (2006). Comparison of odor-active volatile compounds of fresh and smoked salmon. J. Agric. Food Chem..

[5] Varlet V., Serot T., Cardinal M., Knockaert C., Prost C. (2007). Olfactometric determination of the most potent Odor-Active compounds in salmon muscle (*Salmo salar*) smoked by using four smoke generation techniques. J. Agric. Food Chem..

[6] Ojeda M., Bárcenas P., Pérez-Elortondo F.J., Albisu M., Guillén M.D. (2002). Chemical references in sensory analysis of smoke flavourings. Food Chem..

[7] Kelly D., Zerihun A. (2015). The effect of phenol composition on the sensory profile of smoke affected wines. Molecules.

[8] Kennison K.R., Gibberd M.R., Pollnitz A.P., Wilkinson K.L. (2008). Smoke-Derived taint in wine: The release of Smoke-Derived volatile phenols during fermentation of merlot juice following grapevine exposure to smoke. J. Agric. Food Chem..

[9] Hayasaka Y., Parker M., Baldock G.A., Pardon K.H., Black C.A., Jeffery D.W., Herderich M.J. (2013). Assessing the impact of smoke exposure in grapes: Development and validation of a HPLC-MS/MS method for the quantitative analysis of Smoke-Derived phenolic glycosides in grapes and wine. J. Agric. Food Chem..

[10] Wilkinson K.L., Ristic R., Pinchbeck K.A., Fudge A.L., Singh D.P., Pitt K.M., Downey M.O., Baldock G.A., Hayasaka Y., Parker M. (2011). Comparison of methods for the analysis of smoke related phenols and their conjugates in grapes and wine. Aust. J. Grape Wine Res..

[11] Kennison K.R., Wilkinson K.L., Pollnitz A.P., Williams H.G., Gibberd M.R. (2011). Effect of smoke application to Field-Grown merlot grapevines at key phenological growth stages on wine sensory and chemical properties. Aust. J. Grape Wine Res..

[12] Kelly D., Zerihun A., Singh D.P., Von Eckstaedt C.V., Gibberd M., Grice K., Downey M. (2012). Exposure of grapes to smoke of vegetation with varying lignin composition and accretion of lignin derived putative smoke taint compounds in wine. Food Chem..

[13] Krstic M.P., Johnson D.L., Herderich M.J. (2015). Review of smoke taint in wine: Smoke-Derived volatile phenols and their glycosidic metabolites in grapes and vines as biomarkers for smoke exposure and their role in the sensory perception of smoke taint. Aust. J. Grape Wine Res..

[14] Barbosa A., Hogg T., Couto J.A. (2012). The influence of selected oenological practices on the sensory impact of volatile phenols in red wines. J. Int. Des Sci. De La Vigne Et Du Vin.

[15] Fudge A.L., Ristic R., Wollan D., Wilkinson K.L. (2011). Amelioration of smoke taint in wine by reverse osmosis and solid phase adsorption. Aust. J. Grape Wine Res..

[16] Vara-Ubol S., Chambers E., Chambers D.H. (2004). Sensory characteristics of chemical compounds potentially associated with beany aroma in foods. J. Sens. Stud..

[17] Hongsoongnern P., Chambers E. (2008). A lexicon for green odor or flavor and characteristics of chemicals associated with green. J. Sens. Stud..

[18] Wang H., Chambers E. (2018). Sensory characteristics of various concentrations of phenolic compounds potentially associated with smoked aroma in foods. Molecules.

[19] Guillén M.A.D., Manzanos M.A.J. (2002). Study of the volatile composition of an aqueous oak smoke preparation. Food Chem..

[20] Serot T., Baron R., Knockaert C., Vallet J.L. (2004). Effect of smoking processes on the contents of 10 major phenolic compounds in smoked fillets of herring (*Cuplea harengus*). Food Chem..

[21] Noestheden M., Thiessen K., Dennis E.G., Tiet B., Zandberg W.F. (2017). Quantitating Organoleptic Volatile Phenols in Smoke-ExposedVitis vinifera Berries. J. Agric. Food Chem..

[22] Marušić Radovčić N., Vidaček S., Janči T., Medić H. (2016). Characterization of volatile compounds, physico-chemical and sensory characteristics of smoked dry-cured ham. J. Food Sci. Technol..

[23] Chambers E., Koppel K. (2013). Associations of volatile compounds with sensory aroma and flavor: The complex nature of flavor. Molecules.

[24] Buettner A., Beauchamp J. (2010). Chemical input—Sensory output: Diverse modes of Physiology-Flavour interaction. Food Qual. Preference.

[25] Zou Z.H., Buck L.B. (2006). Combinatorial effects of odorant mixes in olfactory cortex. Science.

[26] Ang E.C., Boatright W.L. (2003). Olfactory perception of major odorants found in the headspace of aqueous soy protein isolate slurries. J. Food Sci..

[27] Bott L., Chambers E. (2006). Sensory characteristics of combinations of chemicals potentially associated with beany aroma in foods. J. Sens. Stud..

[28] Zhang B., Cai J., Duan C.-Q., Reeves M., He F. (2015). A review of polyphenolics in oak woods. Int. J. Mol. Sci..

[29] Petričević P., Marušić Radovčić N., Lukić K., Listeš E., Medić H. (2018). Differentiation of dry-cured hams from different processing methods by means of volatile compounds, physico-chemical and sensory analysis. Meat Sci..

[30] Chambers E., Sanchez K., Phan U.X.T., Miller R., Civille G.V., Di Donfrancesco B. (2016). Development of a “living” lexicon for descriptive sensory analysis of brewed coffee. J. Sens. Stud..

[31] Jarma Arroyo S.E., Seo H.-S. (2017). Effects of the type of reference scale on descriptive sensory analysis of cooked rice: Universal aromatic scale versus rice aromatic scale. J. Sens. Stud..

[32] Kim H., Lee J., Kim B. (2017). Development of an initial lexicon for and impact of forms (cube, liquid, powder) on chicken stock and comparison to consumer acceptance. J. Sens. Stud..

[33] Maughan C., Chambers E., Godwin S. (2016). A procedure for validating the use of photographs as surrogates for samples in sensory measurement of appearance: An example with color of cooked turkey patties. J. Sens. Stud..

[34] Talavera M., Chambers D.H. (2016). Flavor lexicon and characteristics of artisan goat cheese from the United States. J. Sens. Stud..

[35] Chang C.Y., Chambers E. (1992). Flavor characterization of breads made from hard red winter wheat and hard white winter wheat. Cereal Chem..

[36] Di Donfrancesco B., Koppel K. (2017). Sensory characterisitcs and volatile components of dry dog foods manufactured with sorghum fractions. Molecules.

